# Novel—and Not So Novel—Inhibitors of the Multifunctional CRM1 Protein

**DOI:** 10.3389/or.2024.1427497

**Published:** 2024-08-05

**Authors:** Waitman K. Aumann, Rafi Kazi, Amanda M. Harrington, Daniel S. Wechsler

**Affiliations:** ^1^ Aflac Cancer and Blood Disorders Center, Children’s Healthcare of Atlanta, Atlanta, GA, United States; ^2^ Department of Pediatrics, Emory University School of Medicine, Atlanta, GA, United States; ^3^ Department of Pediatrics, Division of Pediatric Hematology/Oncology, University of Rochester Medical Center, Rochester, NY, United States; ^4^ Department of Pediatrics, University of Kentucky College of Medicine, Lexington, KY, United States

**Keywords:** CRM1, XPO1, cancer, nuclear export, leptomycin B

## Abstract

Chromosome Region Maintenance 1 (CRM1), also known as Exportin 1 (XPO1), is a protein that is critical for transport of proteins and RNA to the cytoplasm through the nuclear pore complex. CRM1 inhibition with small molecule inhibitors is currently being studied in many cancers, including leukemias, solid organ malignancies and brain tumors. We review the structure of CRM1, its role in nuclear export, the current availability of CRM1 inhibitors, and the role of CRM1 in a number of distinct cellular processes. A deeper understanding of how CRM1 functions in nuclear export as well as other cellular processes may allow for the development of additional novel CRM1 inhibitors.

## Introduction

Chromosome Region Maintenance 1 (CRM1), also known as Exportin 1 (XPO1), is a critical protein involved in the maintenance of normal cellular function. Initially discovered as a protein that is involved in chromosome organization, it has subsequently been found to be involved in a number of different cellular processes responsible for cellular homeostasis, specifically as a protein involved in nuclear export. Studies investigating its role as a nuclear exporter have demonstrated increased expression of CRM1 leads to a worse prognosis in a number of different cancers [1–3]. In addition, mutations of CRM1 and CRM1 fusion partners have been identified in several cancers [4–10]. While a substantial body of work has focused on the nuclear export function of CRM1, it is clear that CRM1 has important functions beyond nuclear export. In order to understand the role of CRM1 in cancer, as well as other physiologic and pathologic processes, we will discuss how unique structural components of the CRM1 protein relate to its diverse roles. We will also review and describe our current understanding of CRM1 inhibition; while this primarily involves inhibition with covalent binders of the C528 residue within the NES cleft, other non-covalent binders of CRM1 are available and may potentially have different side effect profiles from the covalently binding inhibitors. Next, distinct functional, physiologic, and pathologic roles of CRM1 will be summarized, followed by an exploration of mechanisms that control CRM1 protein expression. A deeper understanding of the importance of discrete CRM1 structural domains and their roles in specific cellular functions could lead to the development of novel inhibitors of CRM1.

## CRM1 Structure

**FIGURE 1:**
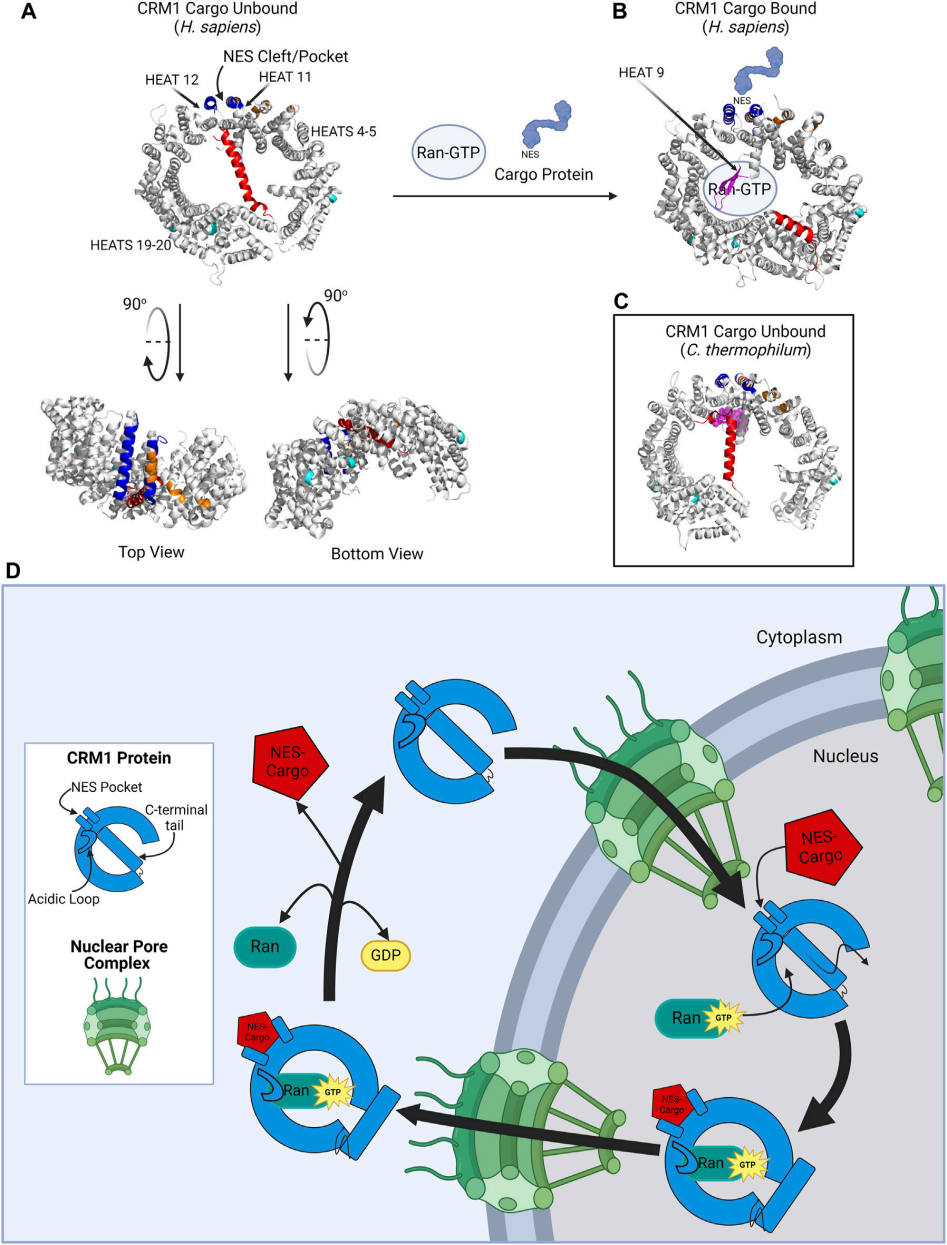
CRM1 structure. **(A)** CRM1 (*H. sapiens*) in its cargo unbound state is donut shaped, with the NES cleft (blue) on the outer convex surface, and the C-terminal tail (red) traversing the hole within CRM1. The acidic loop is not visualized in this structure because of its inability to crystallize in this form; however, the ends of the acidic loops are shown in magenta. The protein has been rotated 90° to show the locations of the dimerization sequence (orange) and the NUP-binding regions (teal). Plumbagin and Oridonin bind pockets between HEAT repeats 4–5 and HEAT repeats 19–20. **(B)** CRM1 (*H. sapiens*) in its cargo-bound form. The NES containing protein (light blue) binds the NES cleft and Ran-GTP binds the internal cavity of the CRM1 protein. The C-terminal tail is then displaced to the periphery of the CRM1 protein. The acidic loop is visualized in this state (magenta) wrapping around GTP. **(C)** CRM1 (*C. thermophilum*) in its unbound state to show the location of the acidic loop (magenta) in close proximity to the NES cleft, as compared to its location in **(B)**. **(D)** Schematic showing CRM1 (blue) shuttling through the nuclear pore complex (NPC, green). In the nucleus, CRM1 undergoes a confirmational change with the simultaneous addition the NES-containing cargo and RanGTP, causing movement of the C-terminal tail, acidic loop, and the NES pocket. The CRM1-RanGTP-Cargo complex transverses the NPC, and upon GTP hydrolysis, Ran and the cargo protein dissociate from CRM1, causing it to go back to its unbound state. Figure created with BioRender.com. Protein structures **(A–C)** from Protein Data Bank (rcsb.org) and modified in PyMol software [11–13].

CRM1 is best known for its role in transporting large proteins or mRNA from the nucleus to the cytoplasm through the nuclear pore. To understand how CRM1 impacts transport, it is critical to understand the CRM1 molecular structure. The CRM1 protein is comprised of 1,071 amino acids that are aligned into 21 HEAT (**H**untingtin, **E**longation factor 3, **A**lpha subunit of protein phosphatase 2A, and the **T**OR1 PI3 kinase). Repeats arranged in antiparallel A and B α-helices (Figure 1). The CRM1 protein forms a donut-shaped structure with the N-terminus of the protein abutting the opposite end of the protein, approximately 45 amino acids from the C-terminus. The terminal 45 amino acids form the C-terminal tail which traverses the ring formed by the protein. The ring shaped CRM1 protein has been found to alternate between two distinct conformations: one bound to a cargo molecule, and one without cargo binding. Due to solubility issues of the unbound CRM1 protein, the CRM1 crystal structure was determined in its cargo-bound shape [14]. The structure of CRM1 was elucidated through X-ray crystallography in the presence of RanGTP and the nuclear import adaptor protein snurportin bound to CRM1 [14]. These studies established that the HEAT repeats of the CRM1 protein form the convex and concave faces of the toroidal structural of CRM1 (Figure 1). Important for nuclear export, HEAT repeats 11 and 12 help form the Nuclear Export Sequence (NES) cleft, where cargo proteins, i.e., proteins bound for nuclear export, bind CRM1 to be shuttled from the nucleus to the cytoplasm [11].

The NES is a sequence of 10–15 hydrophobic, leucine rich amino acids that is present in proteins that are dependent on CRM1 for export from the nucleus to the cytoplasm. The NES of the cargo protein interacts with hydrophobic amino acids located within the NES cleft of CRM1, located between HEAT repeats 11 and 12 on the convex outer surface of CRM1. Cargo proteins, which are too large to simply diffuse across the nuclear membrane, are shuttled through the nuclear pore complex (NPC), with the CRM1-Cargo Protein-RanGTP complex interacting with nucleoporins (NUPs), the proteins of the NPC (Figure 1). Shuttling of proteins via the CRM1 pathway is a highly conserved process: the yeast CRM1 protein shares roughly 70% homology with human CRM1, highlighting the importance of this nuclear export pathway [15].

## Functions of CRM1

### Nuclear Export

The process of cargo binding to the NES cleft within CRM1 has been extensively studied and described [11]. CRM1 undergoes a conformational change when transitioning from its cargo-unbound to its cargo-bound structure (Figure 1). In its native, cargo-unbound state, the NES cleft adopts a closed conformation, whereas when cargo is bound in the NES cleft, the cleft adopts a more open configuration [16]. Concurrently, RanGTP binds to CRM1 due to the conformational change of CRM1’s C-terminal tail and the acidic loop. In its cargo-unbound form, the C-terminal tail (red portion of CRM1 in Figures 1A–C) spans across CRM1, preventing RanGTP binding and modulating the conformation of the CRM1 NES cleft (Figure 1A). In its cargo-bound form, the CRM1 C-terminal tail relocates to the periphery of the protein (Figure 1B). Additionally, in cargo-unbound CRM1, it is suspected that an acidic loop (magenta portion of CRM1 in Figure 1C), a 26-residue stretch between HEAT helices 9A and 9B, interacts with the backside of the NES cleft, whereas when CRM1 is bound to cargo, the acidic loop is removed from this region of the protein [11–13, 17].

There have been two hypotheses proposed for the mechanism by which cargo binds to CRM1: 1) RanGTP binds first with subsequent conformational changes of both the acidic loop and C-terminal tail leading the NES to adopt an open conformation which accepts cargo proteins; 2) alternatively, a cargo protein binds the NES cleft first, leading to conformational changes which permit RanGTP binding. Ultimately, it is most likely that binding of RanGTP and cargo to CRM1 is not a linear process, as Monecke and others have shown cooperative binding where binding of the first molecule enhances affinity for binding of the second, irrespective of which binds first [11, 17, 18]. Upon binding of the cargo protein and RanGTP, CRM1 transforms to a toroidal structure with its N- and C-terminal ends coming together around RanGTP, with the NES-containing cargo attached to the NES cleft. The CRM1-RanGTP-cargo moiety then traverses the nuclear pore complex to enter the cytoplasm, where RanGTP is hydrolyzed to RanGDP, cargo protein is released, and the now cargo-unbound CRM1 can traverse back through the NPC to the nucleus. Cycling of CRM1 between the nucleus and cytoplasm is maintained through a GTP gradient with an increased concentration of GTP in the nucleus [12, 19].

### CRM1 as a Nuclear Export Protein

In its role as a nuclear export protein, CRM1 plays a critical role in maintaining cellular homeostasis as it coordinates the nuclear and cytoplasmic localization of critical proteins in the cell; indeed, CRM1 knockdown leads to cell death, indicating the necessity of CRM1 for development [20]. Additionally, overexpression of CRM1 leads to increased shuttling of proteins from the nucleus to the cytoplasm, disturbing nuclear/cytoplasmic protein balance. Overexpression of CRM1 is seen in numerous malignancies, including leukemias, lymphomas, neuroblastomas, gastric, pancreatic, melanoma, and lung cancers, and is often associated with poor prognosis [21–28]. While the mechanisms of oncogenesis are different for each of these cancers, a common finding is abnormal intracellular shuttling of proteins that leads to tumor propagation. Intriguingly, there are no reports of decreased CRM1 expression in cancers.

### Impact of CRM1 Mutations on CRM1 Function

In order to determine critical regions of the CRM1 protein, multiple groups have analyzed the effect of mutating specific residues the CRM1 protein. The first of these studies evaluated the C-terminal tail, given its role in stabilizing the NES in a closed confirmation. Deletion of the C-terminal tail resulted in a higher affinity of NES-containing cargoes for the CRM1 protein [11, 12, 16, 29]. Furthermore, deletion of the CRM1 C-terminal tail in a CRM1-AF10 fusion oncoprotein induced a more potent murine leukemia in mouse models when compared to a CRM1-AF10 fusion without the C-terminal tail deletion [4]. Of note, a similar deletion has been described in a patient with a *CRM1-AF10* fusion, emphasizing the relevance of CRM1 to cancer pathogenesis [5]. Within the CRM1 C-terminal tail, it has also been found that phosphorylation of the serine at position 1,055 by STK38 (Serine/Threonine Kinase 38) is required for nuclear shuttling, as well as YAP pathway activation, suggesting an important role for this region within the C-terminal tail [30].

The acidic loop on HEAT Repeat 9 (magenta in Figure 1) of CRM1 located across from the NES cleft helps to stabilize the CRM1 protein in a closed conformation, as seen in Figure 1C [11–13]. When RanGTP binds to the CRM1 protein, the position of the acidic loop changes to wrap around RanGTP, assisting the conformational changes that lead to the opening of the NES pocket. Previous work has shown that mutation of the acidic loop region (^430^VLV^432^ to ^430^AAA^432^) enhances the affinity of CRM1 for NES-containing cargo in a manner similar to the deletion of the C-terminal tail [31]. While there were no significant differences in crystal structures of a double mutant involving the C-terminal tail and the acidic loop compared to the C-terminal tail truncation alone [11], when the acidic loop mutations are combined with the C-terminal tail truncated mutant, there is a 12-fold increase in binding strength of cargo to the NES cleft [31].

### CRM1 and Interaction With the Nuclear Pore

During nuclear export, CRM1 directly interacts with the NUPs that make up the nuclear pore. Early work showed that a single antibody identified numerous NUPs; it was later discovered that this antibody was specific for repeated Phenylalanine and Glycine (FG) residues that are found in a subset of NUPs [32]. Subsequent studies showed that FG repeats are necessary for nuclear transport and identified a direct interaction between NUP proteins and CRM1. Further, it was shown that mutations of the CRM1 NUP binding site and mutations of the CRM1 binding site within the Nup82 protein shared a similar phenotype of nuclear localization of ribosomal proteins, which normally reside in the cytoplasm. It was subsequently found that introduction of mutations in the complementary areas of CRM1 or the FG-NUPS abrogated interaction between the proteins [13]. Further, a CRM1-AF10 fusion protein in which the FG-binding regions of CRM1 are mutated no longer induces murine leukemia [4]. Other fusion proteins with NUP moieties, such as SET-NUP214, SQSTM1-NUP214, NUP98-HOXA9, NUP98-DDX10, and NUP98-IQCG have also been shown to interact with CRM1, suggesting the importance of the NUP-CRM1 interaction in these leukemias [33–36].

Additional studies have shown that Nup62, Nup153, and NUP214 colocalize with the protein SUN1 at the NPC [37]. SUN1, and its paralog SUN2, interact with KASH-domain containing proteins found inside the nuclear lamina. These interactions ultimately form a bridge from the nucleoskeleton to the cytoskeleton through a complex known as LINC (Linker of Nucleoskeleton and Cytoskeleton, reviewed in Ref. [38]). Co-immunoprecipitation analysis showed that CRM1 also interacts with SUN1 and SUN2, suggesting that CRM1 is also involved in the LINC complex [39].

Overall, these studies show the importance of the structure-function relationship of the CRM1 protein. The multiple domains of CRM1—the C-terminal tail, the NES pocket, the FG-binding regions–are critical for the process of CRM1-dependent export of cargo through the nuclear pore.

## Inhibition of CRM1

### Covalent Inhibition of CRM1

#### Leptomycin B

**FIGURE 2:**
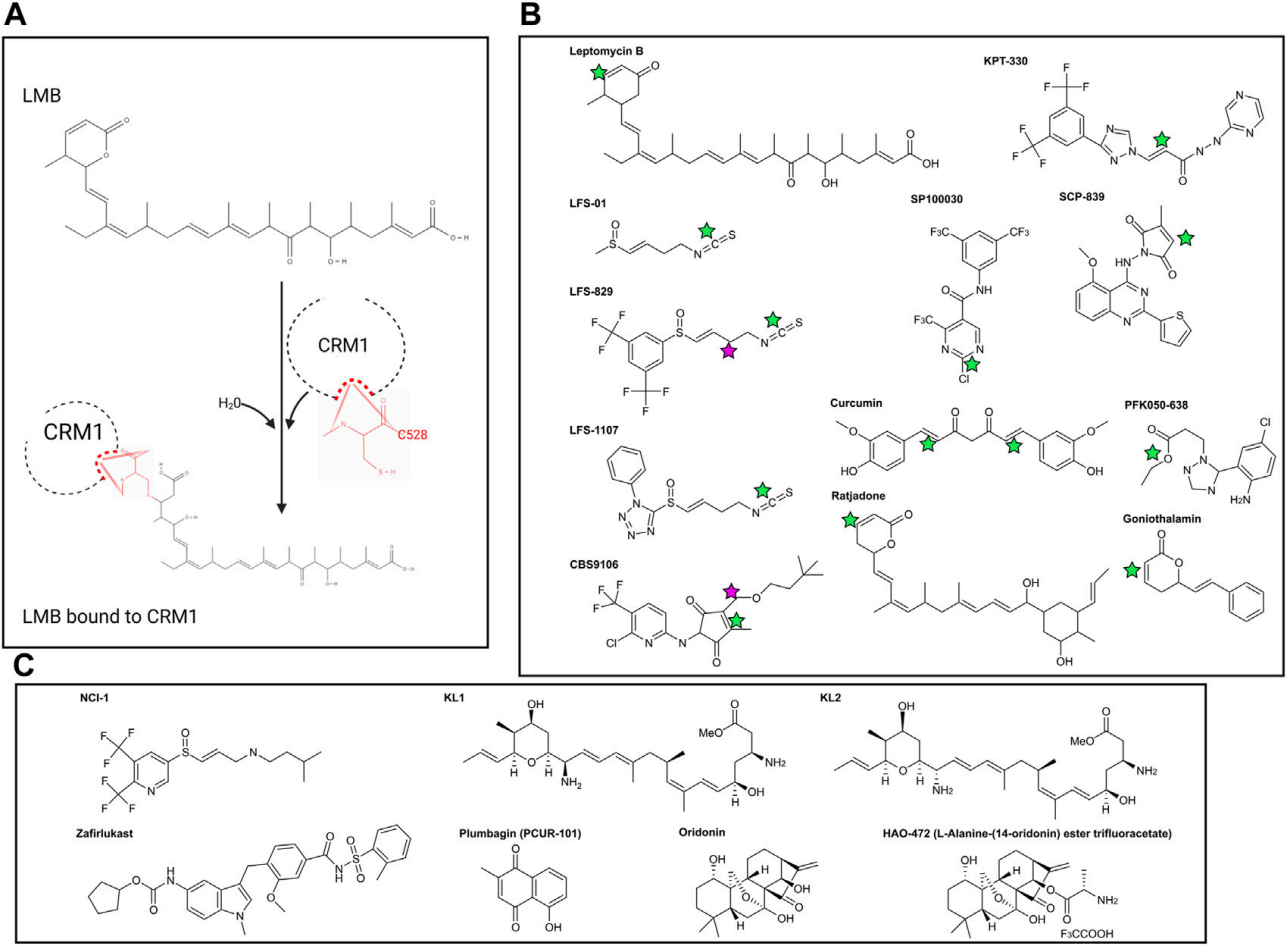
**(A)** Reaction of Leptomcyin B (LMB) with the cysteine residue at CRM1 position 528. With the addition of water, a Michael Reaction occurs between the sulfhydryl group within C528 and the α, β unsaturated lactone within LMB. Note that the hydrolysis reaction causes disruption of the lactone ring within LMB. **(B)** Chemical structures of covalently bonding CRM1 inhibitors. Green stars are the locations of the active α, β unsaturated lactone which forms the covalent bond with CRM1 C528. NCI-1 does not form a covalent bond with C528 and as such does not have the α, β unsaturated lactone. Pink stars in CBS9106 and LFS-829 indicate the site of fusion between the compounds to make NCI-1. **(C)** Chemical structures of non-covalently bonding CRM1 inhibitors. Chemical structures made with ChemDraw.

Inhibition of CRM1 was first discovered through physical blockade of the CRM1 NES cleft by the compound Leptomycin B (LMB). LMB (Figure 2A) was initially discovered as an antifungal agent and later found to have anti-cancer activity [40] as a result of LMB’s direct interaction with the CRM1 NES cleft. The unsaturated lactone ring of LMB undergoes a Michael addition from the nucleophilic side chain of the cysteine at position 528 (C528) within CRM1 (Figure 2A). Hydrolysis of the lactone ring within LMB upon binding to CRM1 results in formation of an irreversible covalent bond between LMB and CRM1. The covalently bonded LMB molecule then blocks any cargo from binding the NES cleft of CRM1, preventing CRM1 from transporting cargo from the nucleus to the cytoplasm. Since numerous cancers require cytosolic localization of proteins (e.g., NF-κB, p53, FOXO1, pRB), blocking the ability of the proteins to enter the cytoplasm results in increased nuclear accumulation, culminating in activation of the apoptosis pathway and leading to death of the cancer cell [41, 42]. While this is an effective mechanism for apoptosis of cancer cells, it also leads to apoptosis of non-malignant cells resulting in significant clinical side effects, including nausea, vomiting anorexia and malaise. These side effects prevented the clinical development of LMB (Elactocin) beyond the initial Phase I clinical trial [43].

#### Selective Inhibitors of Nuclear Export (SINEs)

Selective Inhibitors of Nuclear Export (SINEs) have been developed as alternatives to LMB to inhibit nuclear export by blockade of CRM1 (Figure 2B). Both LMB and the SINEs form a covalent bond with the C528 [44]; however, unlike LMB, there is no hydrolysis of a lactone ring within the SINEs, allowing for the SINEs to reversible bind to CRM1 [45]. The SINEs have been shown to have a similar effect of increasing nuclear localization of cytoplasmic proteins, but importantly, their side effects in patients have been substantially less than those seen with LMB. Promising results for trials using SINEs in Phase I, II, and III clinical trials have been published for acute leukemias, glioblastoma, multiple myeloma, non-Hodgkin lymphoma, and ovarian cancers, and numerous other trials using SINEs are ongoing (Tables 1, 2) [46–51]. In addition, SINEs appear to have efficacy in a number of different malignancies, including hematologic, epithelial, and mesenchymal tumors in the pre-clinical setting [42, 52–55]. Finally, while initial studies used SINEs as single agents, more recent work has shown synergy between SINEs and other chemotherapy agents, including steroids, venetoclax, ibrutinib, cisplatin, carfilzomib, doxorubicin, gemcitabine, and azacitidine [27, 56–66].

**TABLE 1:** Active trials using CRM1 inhibitors.

Disease grouping	Disease	Study number	Study phase	Status	CRM1 inhibitor	Concurrent therapy
Hematologic Malignancy	Acute Lymphoblastic and Acute Myeloid Leukemia	NCT02091245	Phase 1	Active, Not Recruiting	Selinexor	
	Acute Myeloid Leukemia	NCT02835222	Phase 2	Active	Selinexor	Cytarabine, Daunorubicin
	Acute Myeloid Leukemia	NCT05736965	Phase 2	Active	Selinexor	Azacitidine, Venetoclax
	B Cell Non-Hodgkin’s Lymphoma	NCT03147885	Phase 1b/2	Active	Selinexor	Rituximab, Cyclophosphamide, Doxorubicin, Vincristine, Prednisone
	DH or TH Lymphoma	NCT05974085	Phase 2	Active	Selinexor	Rituximab, Cyclophosphamide, Doxorubicin, Vincristine, Prednisone
	Diffuse Large B-Cell Lymphoma	NCT05422066	Phase 2	Active	Selinexor	Rituximab, Cyclophosphamide, Doxorubicin, Vincristine, Prednisone
	Diffuse Large B-Cell Lymphoma	NCT05577364	Phase 1b/2	Active	Selinexor	Rituximab, Cyclophosphamide, Doxorubicin, Vincristine, Prednisone
	Diffuse Large B-Cell Lymphoma	NCT02227251	Phase 2	Active	Selinexor	
	High Risk Hematologic Malignancies	NCT03955783	Phase 1	Active, Not Recruiting	Selinexor	Venetoclax
	Multiple Myeloma	NCT04877275	Phase 2	Active	Selinexor	Pegylated liposomal doxorubicin, Dexamethasone, Cyclophosphamide
	Multiple Myeloma	NCT04891744	Phase 1b/2	Active, Not Recruiting	Selinexor	Thalidomide, Dexamethasone
	Multiple Myeloma	NCT04519476	Phase 1	Active	Selinexor	Lenalidomide, Methyprenisolone
	Multiple Myeloma	NCT06225310	Phase 1	Active, Not Recruiting	Selinexor	Ruxolitinib, Methylprednisolone
	Multiple Myeloma	NCT05422027	Phase 1/2	Active	Selinexor	Bortezomib, Lenalidomide, Dexamthasone
	Multiple Myeloma	NCT02199665	Phase 1	Active	Selinexor	Carfilzomib, Dexamethasone
	Multiple Myeloma	NCT04941937	Phase 2	Active	Selinexor	Thalidomide, Lenalidomide, Pomalidomide, Dexamethasone
	Multiple Myeloma	NCT04756401	Phase 2	Active	Selinexor	Carfilzomib, Daratumumab, Dexamethasone
	Multiple Myeloma	NCT04764942	Phase 1/2	Active	Selinexor	Carfilzomib, Dexamethasone, Pomalidomide
	Multiple Myeloma	NCT04925193	Phase 2	Active	Selinexor	Carfilzomib, Dexamethasone, Pomalidomide
	Multiple Myeloma	NCT06169215	Phase 2	Active	Selinexor	Daratumumab, Bortezomib, Dexamethasone
	Multiple Myeloma	NCT04877275	Phase 2	Active	Selinexor	Doxil, Dexamethasone, Cyclophosphamide
	Multiple Myeloma	NCT06225310	Phase 1	Active, Not Recruiting	Selinexor	Ruxolitinib, Methylprednisolone
	Multiple Myeloma	NCT05597345	Phase 2	Active	Selinexor	
	Myelodysplastic Syndromes	NCT05918055	Phase 1/2	Active	KPT-8602	Decitabine-Cedazuridine
	Myelodysplastic Syndromes and Acute Myeloid Leukemia	NCT06399640	Phase 1	Active, Not Recruiting	KPT-8602	Venetoclax
	Non Hodgkin or Hodgkin lymphoma or histiocytic/dendritic cell neoplasm	NCT04640779	Phase 1	Active	Selinexor	Choline Salicylate
	Peripheral T-cell Lymphomas	NCT05822050	Phase 2/3	Active	Selinexor	
Non-CNS Solid Tumor	Endometrial Carcinoma	NCT05611931	Phase 3	Active	Selinexor	Placebo-Controlled
	Endometrial Carcinoma	NCT03555422	Phase 3	Active, Not Recruiting	Selinexor	Placebo-Controlled
	Recurrent and Refractory Pediatric Solid Tumors, Including CNS Tumors	NCT02323880	Phase 1	Active, Not Recruiting	Selinexor	
	Alveolar Soft Part Sarcoma	NCT05333458	Phase 2	Active	Selinexor	Atezolizumab
	Breast Cancer	NCT05035745	Phase 1/2	Active	Selinexor	Talazoparib
	Gastrointestinal Stromal Tumors	NCT04138381	Phase 1/2	Active, Not Recruiting	Selinexor	Imatinib
	Melanoma	NCT04768881	Phase 2	Active, Not Recruiting	Selinexor	Pembrolizumab
	Non-Rhabdomyosarcoma Soft Tissue Sarcoma	NCT06239272	Phase 1/2	Active	Selinexor	Pazopanib, Ifosfamide, Doxorubicin
	Ovarian, Fallopian or Primary Peritoneal carcinoma	NCT05983276	Phase 2	Active	Selinexor	Decitabine, Carboplatin, Paclitaxel
	Soft Tissue Sarcomas	NCT04811196	Phase 1	Active, Not Recruiting	Selinexor	
	Soft-tissue Sarcoma and Osteosarcoma	NCT04595994	Phase 1/2	Active	Selinexor	Gemcitabine
	Solid Neoplasm	NCT02419495	Phase 1	Active, Not Recruiting	Selinexor	Based on disease type
	Urothelial Carcinoma	NCT04856189	Phase 1b/2	Active	Selinexor	Pembrolizumab
	Urothelial Carcinoma	NCT04856189	Phase 1/2	Active	Selinexor	Pembrolizumab
	Wilms Tumor, Rhabdoid Tumor, MPNST	NCT05985161	Phase 2	Active	Selinexor	
CNS Tumor	Diffuse Intrinsic Pontine Glioma or High-Grade Glioma	NCT05099003	Phase 1/2	Active	Selinexor	Temozolomide
Other	Relapsed/Refractory Cancer	NCT02649790	Phase 1/2	Active, Not Recruiting	KPT-8602	Decitabine-Cedazuridine, Dexamethasone

**TABLE 2:** Inactive trials that used CRM1 inhibitors.

Disease grouping	Disease	Study number	Study phase	Status(as of 6/3/24)	CRM1 inhibitor	Concurrent therapy
Hematologic Malignancy	Acute Myeloid Leukemia	NCT02093403	Phase 1	Closed	Selinexor	Decitabine
	Acute Myeloid Leukemia	NCT02299518	Phase 1	Closed	Selinexor	Etoposide, Mitocantrone, Cytarabine
	Acute Myeloid Leukemia	NCT02249091	Phase 2	Closed	Selinexor	Idarubicin, Cytarabine
	Acute Myeloid Leukemia	NCT02530476	Phase 1/2	Closed	Selinexor	Sorafenib
	Advanced Hematological Cancer	NCT01607892	Phase 1	Closed	Selinexor	
	AML and MDS	NCT02485535	Phase 1	Closed	Selinexor	
	B-Cell Lymphoma	NCT02471911	Phase 1	Closed	Selinexor	Rituximab, Etoposide, Carboplatin, Ifosfamide, Dexamethasone
	Multiple Myeloma	NCT06212596	Phase 2	Closed	Selinexor	Cyclophosphamide, Prednisone
	Myelodysplastic Syndromes	NCT02228525	Phase 2	Closed	Selinexor	
Non-CNS Solid Tumor	Small Cell Lung Cancer	NCT02351505	Phase 2	Closed	Selinexor	
	Advanced Gynecologic Malignancies	NCT02025985	Phase 2	Closed	Selinexor	
	Advanced Solid Tumors	NCT02667873	Phase 1	Closed	SL-801	
	Breast Cancer	NCT02402764	Phase 2	Closed	Selinexor	
	Gastric, Gastro-esophageal junction or Distal Esophageal Adenocarcinoma	NCT02283359	Phase 1	Terminated	Selinexor	Irinotecan
	Liposarcoma	NCT02606461	Phase 2/3	Closed	Selinexor	Placebo-Controlled
	Lung and Gastroenteropancreatic Tumors	NCT02250885	Phase 2	Closed	Selinexor	
	Melanoma	NCT02120222	Phase 1	Closed	Selinexor	
	Non-small Cell Lung Cancer	NCT03095612	Phase 1/2	Closed	Selinexor	Docetaxel
	Pancreatic Cancer	NCT02178436	Phase 1b/2	Closed	Selinexor	Gemcitabine Hydrochloride, Nab Paclitaxel
	Prostate Cancer	NCT02215161	Phase 2	Closed	Selinexor	
	Soft Tissue Sarcomas	NCT03042819	Phase 1	Closed	Selinexor	Doxorubicin
	Solid Neoplasm	NCT01607905	Phase 1	Closed	Selinexor	
	Squamous Cell Lung Cancer	NCT02536495	Phase 1/2	Withdrawn (funding support)	Selinexor	Docetaxel

While SINEs have been extensively used in numerous cancers, recognition of the downstream effects of this class of drugs continues to be studied. For instance, it has been shown that treatment with SINEs is effective in decreasing HOX/MEIS1 activation and prolonged survival of mice harboring a mutated NPM1 protein, which directly binds CRM1 [67]. Interestingly, while wildtype NPM1 does not contain a nuclear export sequence, a recurrent mutation (seen in one-third of adult AML, and 6.5% of childhood AML) results in appearance of a novel NES within the NPM1 protein, resulting in mislocalization of the NPM1 protein to the cytoplasm [68, 69]. It is thought that SINEs, by directly binding to the CRM1 NES cleft, block CRM1-mediated export of mutated NPM1, which induces nuclear localization of mutated NPM1, thereby restoring the cell to its normal homeostasis. While the efficacy of SINEs in NPM-mutated leukemias implicates CRM1 in leukemogenesis, further mechanisms underlying the NPM/CRM1 interaction have yet to be revealed. Indeed, early clinical trials using KPT-330, a second-generation SINE, have shown some promise, but the clinical effect has been somewhat muted compared to preclinical data. One possibility is that decreased treatment frequency (2 days a week) due to adverse events led to reduced efficacy. Subsequent third generation SINEs have shown increased tolerability despite increased dosing frequency, and these will be studied in upcoming clinical trials [70]. Because of the interaction of NPM1/CRM1 with centrosomes (see below), future investigations could focus on disruption of centrosome formation in AML, and potentially other cancers.

#### Sulforaphene and Its Derivatives

Sulforaphene (also known as LFS-01) (Figure 2B), the major active compound in *Lai Fu Zi* (*Raphanus sativus*) which has been used in traditional Chinese medicine for over one thousand years, was shown to covalently bind the cysteine at position 528 in CRM1. While LFS-01 does not contain the unsaturated lactone present in LMB and SINEs, it does contain an isothiocyanate group which allows for a similar Michael Reaction to occur, allowing for covalent bonding of LFS-01 to the cysteine at position 528 of CRM1 (71). Structure-based discovery models were used to modify LFS-01 and led to the creation of LFS-829, which includes a ditrifluoromethy-phenyl group [71]. This moiety, which is also found in the SINE compound KPT-330, allowed for stronger non-covalent binding of the compound in the NES cleft as well as increased potency compared to its predecessor. The group then knocked down CRM1 via siRNA in HCT-15 cells and showed that treatment with LFS-829 was not as effective compared to the wild-type cell line, indicating LFS-829 targets CRM1. They further showed that LFS-829 and KPT-330 have similar binding to CRM1 (K_d_ ∼ 26.95 nM for LFS-829 and K_d_ ∼ 18.74 nM for KPT-330) with LFS-829 showing more reversibility evidenced by less nuclear accumulation of GFP in LFS829 treated 293T cells 2 h after washout compared to KPT-330 treated 293T cells. Mechanistically, LFS-829 induced nuclear retention of IκB_α_, which inhibits NF-κB transcriptional activation, a pattern seen in other CRM1 inhibitors discussed below. *In vivo* murine work showed no toxic effects of lethargy, anorexia, or other indicators of physical illness of LFS-829 in mice treated with 300–600 mg/kg over the course of 1 day. Current work with LFS-829 has been limited to pre-clinical studies in inflammatory bowel diseases (IBD) and there are no reports of clinical trials.

The same group has developed a follow up compound to LFS-829, LFS-1107, using a deep reinforcement learning molecular *de novo* design method to identify structures bearing similarity to the parent compound [72]. Similar to its parent compound and synthetic analogue, LFS-1107 also binds the NES-cleft within CRM1, with a stronger affinity for CRM1 than KPT-330 (K_d_ ∼ 0.0125 nM compared to K_d_ ∼ 5.29 nM). Correspondingly, the IC_50_ for LFS-1107 in triple-negative breast cancer (TNBC) is lower (40.80 nM) than the IC_50_ for KPT-330 (69.47 nM). LFS-1107 specifically slowed growth of four TNBC cell lines compared to normal breast cancer epithelial cells. Additionally, the authors show that LFS-1107 may also reverse drug resistance in the TNBC cell lines through downregulation of the multidrug resistant proteins ABCB1 and ABCG2. The *in vitro* results of LFS-1107 translated to murine *in vivo* work showing that TNBC xenograft tumors treated with LFS-1107 for 10 days compared to the vehicle treated group; notably the mice had no changes in body weight during the treatment course, indicating the drugs relative safety [72]. The same group then also published that LFS-1107 also suppresses the growth of extranodal NK/T-cell Lymphoma (ENKTL), a notably difficult lymphoma to treat. In addition to showing efficacy *in vitro*, the group shows that LFS-1107 prolongs survival and reduces spleen weight of SCID mice injected with ENKTL cells [73]. Similar to LFS-829, there are no clinical trials at present using LFS-1107. Given the increased strength of binding of LFS-1107 to CRM1, questions remain as to the reversibility of this compound, compared to both its analogue LFS-829 and KPT-330. Future studies investigating the sulforaphene analogues may provide an alternative to KPT-330 with potentially fewer side effects.

#### CBS9106/Felezonexor

CBS9106 (Figure 2B) was found to be a CRM1 inhibitor through the use a chemical library screen. Initially found to have IC_50_ values in the nanomolar range for 60 human cancer cell lines, it was found that CBS9106 acted similarly to LMB in its ability to retain RanBP1 in the nucleus. Similar to the sulforaphenes and LMB, CBS9106 appears to inhibit NF-κB activity through prevention of IκB_α_ degradation. Unlike the previously discussed inhibitors, CBS9106 causes a decrease the amount of CRM1 protein in cancer cells, without a compensatory change in the amount of CRM1 mRNA, leading the group to hypothesize that CBS9106 induces CRM1 protein degradation through ubiquitin/proteosome pathway, a hypothesis substantiated by the absence of CRM1 degradation upon the addition of two separate proteosome inhibitors (bortezemib and MG132). Interestingly, the authors further show that in addition to activation of the ubiquitin/proteosome pathway, CBS9106, like LMB, binds to CRM1 at Cys528, though in a reversible nature, indicating that CBS9106 has further activity than blockade of the NES cleft. This further activity was elaborated by the same group which showed CBS9106 induces neddylation of CRM1, leading to ubiquitination and therefore degradation of CRM1 through the proteasomal pathway [74]. This was shown with the use of MLN4924 (Pevonedistat), a neddylation inhibitor which has recently been shown to safe in a Phase I clinical trial in combination with azacytidine and venetoclax in relapsed/refractory AML [75]. When CBS9106 was combined with MLN4924, the reduction in the amount of CRM1 protein was attenuated. Further, the use of MLN4924 blocked the inhibitory effects of CBS9106 on nuclear localization; that is, nuclear localization of proteins induced by CBS9106 was negated with MLN4924. This indicates that CBS9106’s activity on CRM1 inhibition, at least in part, occurs through the neddylation pathway, which was further shown by knockdown of proteins NEDD8, UBA3, or Rbx1 showing similar effects to MLN4924 [74]. *In vivo* work showed a dose response effect of CBS9106 on tumor growth and mouse survival in multiple myeloma xenografts [76]. CBS9106, also known as felezonexor, was used in a phase I trial (NCT02667873) which closed in 2022; preliminary results showed safety and efficacy in population of advanced and heavily pre-treated solid tumors. In this group of 57 patients assessed prior to trial completion, 1 patient with colorectal cancer had a partial response, while 14 patients had stable disease [77].

#### Selective Inhibitors of Transcriptional Activation (SITAs)

Recently published work identified two additional CRM1 inhibitors that also bind C528 within the NES-cleft of CRM1, SP100030 and SPC-839 (Figure 2B) [78]. Similar to the SINE class of small molecule inhibitors, these drugs are active against wild-type CRM1, however inactive in cells harboring the C528S mutation. Interestingly, while both the SINE inhibitors and SP100030/SPC-839 decrease IL-2 activity in a similar manner, the cytotoxic effects of the SINEs are much higher than SP100030 and SPC-839. The authors thus hypothesize that SP100030 and SPC-839 have an alternative effect on CRM1, and labelled this class of inhibitors Selective Inhibitors of Transcriptional Activation (SITAs).

In the same work, this group showed that CRM1, in addition to its role as a nuclear exporter protein, occupies numerous areas of the genome including genes involved in T-cell activation and differentiation [78–80]. They then showed that treatment with SITAs disrups the CRM1/Chromatin interaction, without impacting nuclear export. While yet to be used clinically, this group did show that the impact on murine bone marrow, was reduced, with decreased neutropenia and thrombocytopenia compared to the SINE-class of inhibitors. Despite this promising result, it can be reasoned that these inhibitors, while seemingly similar to the SINEs, could have a significantly different side effect profile given their ability to dissociate CRM1 from chromatin, without affecting its role in nuclear export.

#### Other Compounds

There are other CRM1 inhibitors that covalently attach to C528 located within the CRM1 NES, similar to LMB and the SINEs. These include synthetic LMB derivatives, the ratjadones, PKF050-638, gonothalium and its derivatives, and curcumin (Figure 2B) [81–87]. The majority of these CRM1 inhibitors contain an unsaturated lactone which serves as the nucleophilic acceptor in the Michael reaction with the cysteine at position 528 in CRM1, and as such, are covalent inhibitors of CRM1.

### Non-Covalent Inhibition of CRM1

#### NCI-1

Using what was presumed to be the active moieties of LFS-829 and CBS9106, the sulfinyl attached ditrifluoromethy-phenyl group and dimethylbutyl group, respectively, Lei et al designed a novel compound that non-covalently binds CRM1 with an increased ability to be reversible known as NCI-1 (Noncovalent CRM1 Inhibitor −1) that showed an ability to inhibit nuclear export (Figure 2C) [88]. Further, inhibition of nuclear export was maintained despite endogenous CRM1 harboring a C528S mutated-CRM1 (CRM1^C528S^) indicating that NCI-1 does not covalently bind the sulfhydryl group of the cysteine at position 528 in CRM1 (88). Thus, this compound, which was created through the combination of two separate CRM1 inhibitors, allows for reversible CRM1 inhibition without covalent modifications, potentially resulting in decreased adverse events when used clinically. Unfortunately, this compound is not stable in the presence of DTT dampening its excitement for clinical use [89].

#### Zafirlukast

The same group then used a virtual screen to look for compounds that bound CRM1, and determined that Zafirlukast (Figure 2C), a leukotriene receptor antagonist that is FDA approved for asthma, bound the NES of CRM1 in pull-down experiments. Intriguingly, unlike the SINEs and other covalent CRM1 binding inhibitors, and like NCI-1, Zafirlukast maintained CRM1 inhibition in the presence of the C528S mutation within CRM1. As an improvement on NCI-1, Zafirlukast is not degraded in the presence of DTT. Modifications of the Zafirlukast structure, including shifting of the methyl group on the terminal benzene ring of Zafirlukast, deletion of the cycopentyl ring, and methylation of the sulfoamides, showed the importance of these structures for Zafirlukast to non-covalently bind CRM1. Specifically, Zafirlukast has an IC_50_ of 44 μM in a gastric carcinoma cell line, has an on target effect using a thermal shift assay, and synergizes with doxorubicin [89]. While Zafirlukast has been FDA approved for asthma, it has not yet been evaluated clinically for cancer.

NCI-1 and Zafirlukast are the first two compounds that have shown an ability to non-covalently bind to the CRM1 protein. While the IC_50_ for Zafirlukast remains high compared to KPT-330 (in nanomolar range in HGC27 cell line [90]), future derivatives of Zafirlukast may be more potent, allowing for further development of non-covalent inhibitors of CRM1.

#### KL1, KL2

As discussed above, the ratjadones are a class of known inhibitors that covalently bind CRM1. KL1 and KL2 (Figure 2C) are aminoratjadone derivatives that lack the lactone ring, and thus lack the ability to undergo a Michael addition, yet they are able to inhibit CRM1-mediated nuclear export [91]. The chemical modifications, combined with experimental evidence that CRM1 could bind NES following treatment with KL1 or KL2 indicates that these compounds do not covalently bond with the C528 of CRM1, despite maintaining their ability to block nuclear export. Further, this group found that KL1 and KL2 also decreased the amount of nuclear CRM1, which they hypothesized was due to proteosomal degradation, a finding not seen with SINE class of CRM1 inhibitors. Additionally, KL1 and KL2 slowed the growth and induced apoptosis in colorectal cell *in vitro* [91]*.* Together, these results show a potential novel compound that is able to non-covalently bind to CRM1, and allows for further investigations of CRM1 inhibition and degradation.

#### Plumbagin and Oridonin

The naturally occurring compounds, plumbagin and oridonin (Figure 2C), have also demonstrated significant anti-cancer activity with similar nuclear localization of CRM1. Interestingly, crystal structures show that in addition to the compounds localizing in the NES cleft, they also bind two separate pockets in CRM1: one between HEATS 4 and 5, and the other between HEATS 19 and 20 (Figure 1). The authors note that the NES-binding groove was more open compared to when traditionally bound NES cargoes bind, potentially allowing for a coordinated effect of further opening of the NES to allow the bulky compounds into the NES cleft [92]. Finally, it has been shown that oridonin increases the expression of Nup98, one of the nucleoporins noted to interact with FG-repeats within CRM1 [93]. This could indicate that oridonin binds FG repeats, blocking NUP98 binding, resulting in a compensatory increase in Nup98 to enhance nuclear export.

While plumbagin and oridonin interact with CRM1, they both have been shown to be active in a number of different pathways. Specifically, a recent review of plumbagin has shown many different biological activities of plumbagin including its role in inflammation, its cytoprotective activity, and its role in stem cells and cell senescence [94]. Additionally, a recent review of oridonin and cancer describes oridonin, including the source of the plant, general molecular features, with descriptions of its anti-cancer activities *in vitro* and *in vivo*, and potential mechanisms of action [95]. These two reviews highlight the complicated nature of plumbagin and oridonin, as would be expected for plant-derived medicinal agents. Covering these pathways and potential side effects of these drugs is beyond the scope of this review, though we will discuss how these compounds have been used clinically.

PCUR-101 (Figure 2C), a synthetic version of plumbagin was used in a Phase I clinical trial (NCT03037758). This phase I trial found that PCUR-101, combined with androgen deprivation therapy (ADT) in metastatic, castrate resistant prostate cancer, was safe and may prolong disease stability [96]. A second phase I trial to evaluate the maximal tolerated dose was terminated due to lack of enrollment on 10/23/23, with no results available at this time. Another group synthesized a derivative of the oridonin, HAO472 [97], and it currently is in a Phase I clinical trial in China, evaluating its effect in patients with AML (chinadrugtrials.org.cn, trial number CTR20150246). Results of this trial are not available at this time. While this is being used in China, this drug has yet to be approved in the United States, likely in light of its many possible mechanisms of action, and thus potential for adverse effects. As might be expected with plant derivatives, both plumbagin and oridonin are still early in clinical trial efficacy.

## Summary

Studying inhibition of CRM1 with CRM1 inhibitors has been critical in uncovering underlying mechanisms of action of CRM1 as well as the functional roles of CRM1. Inhibition of CRM1, regardless of the exact mechanism, leads to perturbations in the nuclear/cytoplasmic ratio of many proteins, with this perturbation often leading to cellular apoptosis. Indeed, numerous cancer cell lines show evidence of apoptosis following treatment with LMB or one of the SINEs in both *in vitro* and *in vivo* experiments. Additionally, there has been work evaluating synergy between the SINEs with traditional chemotherapy, indicating that CRM1 may have a role in overcoming drug resistance. For instance, the addition of KPT-330 to a proteosome inhibitor had a synergistic effect in multiple myeloma cells *in vitro* that were refractory to the proteosome inhibitor alone [98]. Subsequent clinical trials have evaluated the combination of these agents in refractory multiple myeloma, and this combination has shown have a response in patients with a median survival of 15 months in this heavily pre-treated cohort [99]. Another study showed that the KPT-330 overcomes ibrutunib resistance in mantle cell lymphoma (MCL), through the nuclear retention of IκB [100]. In MCL, ibrutinib causes a downregulation of NFκB, though this is lost in MCL cells resistant to ibrutinib. Upon the addition of KPT-330, the increased nuclear IκB in binds NFκB, causing a downregulation of NFκB, leading to apoptosis. This pre-clinical work served as the foundation for the Phase I trial combining these agents, which showed the combination of drugs to be effective [101]. While there is not a current study evaluating the effectiveness of this combination, one was recently suspended (NCT04607772). In addition to these two agents, more studies are evaluating the combination of KPT-330 with other agents, using the KPT-330’s effect on nuclear localization to the advantage of these agents (Tables 1, 2).

Continued study of the structure of CRM1 as well as the structure of CRM1 inhibitors may lead to the development of novel synthetic compounds allowing for more potent and specific effects, without abrogating normal CRM1 function. While these have not been described, other potential targets could be interrupting the protein-protein interactions between CRM1 and NUP proteins, interfering with RanGTP binding, interfering with C-terminal tail displacement or the movement of the acidic loop during RanGTP/NES-cargo binding, or the synthesis of other allosteric inhibitors that alter the conformation of CRM1 in either its NES-cargo bound or unbound state. With a multi-pronged approach, the activity of CRM1 in cancer cells may be mitigated while allowing for maintenance of its physiologic role in non-malignant cells.

## CRM1 and Chromosomal Organization

In addition to nuclear export, CRM1 has a number of other cellular roles. Indeed, the protein’s original name, Chromosome Region Maintenance 1 (CRM1), was derived from the fact that mutations within CRM1 led to disordered chromosomal superstructure [102]. Furthermore, cells with wild-type CRM1 showed normal development of a single centromere, whereas cells with mutated CRM1 had multiple centromeres leading to abnormal chromosomal separation [103].

CRM1 is important in the formation of centrosomes. During mitosis, many proteins, including CRM1, are involved in regulation of centrosome duplication to allow for cell division (reviewed in [104]). The necessity of CRM1 for normal centrosome duplication was shown by using the CRM1 inhibitor Leptomycin B (LMB): there was a positive correlation between LMB dose and percentage of mitotic cells with abnormal duplication of centrosomes [105]. Further work on the involvement of CRM1 with the centrosome showed that overexpression of CRM1 led to inhibited spindle assembly with premature microtubule attachment to the kinetochores which led to abnormal separation of the chromosomes during mitosis [106, 107].

Another mechanism of abnormal centrosome duplication in the presence of LMB involves Nucleophosmin1 (NPM or NPM1), which harbors an NES in the N-terminal region of the protein. When NPM1 and CRM1 are bound together through the NPM1 NES, the NPM1/CRM1 complex associates with a single centrosome; notably, NPM1 does not associate with centrosomes in cells during interphase, i.e., when cells have two centrosomes. Thus, as part of the tightly controlled centrosome duplication process, it is thought that NPM1 is a negative regulator of centrosome duplication. In early mitosis, the block on centrosome duplication is removed through NPM1 phosphorylation by the cell cycle proteins CDK2/cyclin E phosphorylase, which causes dissociation of NPM1 and the centrosome [108]. The ability of NPM1 to negatively regulate centrosome duplication suggests that NPM1 knockout will result in centromere duplication. Correspondingly, exposure to the CRM1 inhibitor LMB, which blocks the ability of CRM1 to bind to NPM1, also results in abnormally increased centrosome duplication. The underlying mechanism is likely due to inhibition of the interaction between CRM1 and NPM1, since an NES-mutated NPM1 could not rescue NPM1-silenced cells, while a wildtype NPM1 could [109]. Thus, CRM1 potentially serves as a bridge between the centrosome complex and NPM1. However, it remains unknown how the CRM1/NPM1 complex localizes to the centrosome. One possibility is that CRM1 may form a complex with components of the centrosome (HOPS, y-tubulin and eEF-1A) in the cell cytosol just prior to mitosis [110]. Thus, CRM1 appears to be involved not only in chromosomal segregation, but also in microtubule organization during the cell cycle, all pointing back to its original discovery as a protein in involved in maintenance of chromosomes.

Taken together, it appears that CRM1 plays an important role in the organization of chromosomes during mitosis, a process that is affected by two of the CRM1 inhibitors addressed above (Leptomycin B and KPT-330) when used *in vitro*. Future studies should evaluate the role of these inhibitors in cancer cells to determine if a mechanism of cell death is SINE-induced nuclear retention of CRM1, leading to microtubule, kinetochore, and centrosome dysregulation, and thus activation of the apoptotic pathway. Further understanding of CRM1 upregulation in non-malignant cells may also help us better understand the mechanism of the adverse events of the SINEs seen clinically such as anorexia, nausea, and vomiting.

## CRM1 and Viral Pathogenesis

CRM1 has been shown to play a role in viral processes including RNA export and viral replication, with early Human Immunodeficiency Virus-1 (HIV-1) studies demonstrating the importance of CRM1 in RNA export. Through a mechanism similar to that described above for nuclear export, the Rev protein, encoded by HIV-1, binds the CRM1 NES cleft through NES sequences within Rev in a RanGTP-dependent mechanism [111, 112]. In addition to binding CRM1, Rev also binds the Rev Response Element (RRE), which is a complex of HIV RNAs, and then the CRM1-Rev-RRE complex traverses the NPC, translocating RNAs from the nucleus to the cytoplasm [113].

Further HIV-1 nuclear export studies have shown that CRM1 directly interacts with RNA Binding Motif Protein 14 (RBM14, also known as PSP2, para-speckle protein 2), which is a cofactor for the HIV-1 Rev protein [114]. RBM14 expression positively correlates with Rev activity, and it is necessary for HIV viral replication and is involved in the export of unspliced viral RNA. RBM14 plays an essential role in the maintenance of genome integrity in mouse embryo development and has also been found in nuclear paraspeckles [115]. These nuclear paraspeckles contain the long non-coding lncRNAs *NEAT1* and *NEAT2*, as well as a heterodimer of nuclear factors p54nrb and PSF (polypyrimidine tract-binding protein-associated splicing factor). p54nrb and RBM14 both interact with CRM1, indicating likely CRM1 involvement in the paraspeckle. Paraspeckle formation, and in turn activity of RBM14, however, is reliant upon the lncRNA *NEAT1*, with knockdown of *NEAT1* leading to dissolution of the paraspeckle [114]. While the specific role of CRM1 in this process is not clearly defined, based on its interactions with multiple proteins in the paraspeckle, further investigations are warranted.

CRM1 also contains a homodimerization sequence (shown in yellow in Figure 1), and during viral HIV export, two CRM1 proteins combine with Rev and the RRE [116]. Intriguingly, the homodimerization sequence consists of seven amino acids that are not conserved between murine and human proteins, and murine CRM1 does not homodimerize (Table 3). The inability of murine CRM1 to form a dimer has been postulated to reduce the ability of murine cells to support HIV replication [117], which may be a reason mouse models are inadequate for studying HIV [118].

**TABLE 3:** Human vs. murine amino acids in the dimerization sequence of CRM1.

CRM1 position	Human CRM1 residue	Murine CRM1 residue
346	Threonine	Alanine
411	Proline	Threonine
412	Methionine	Valine
414	Phenylalanine	Serine
474	Arginine	Isoleucine
478	Glutamic acid	Lysine
481	Histidine	Glutamine

**FIGURE 3:**
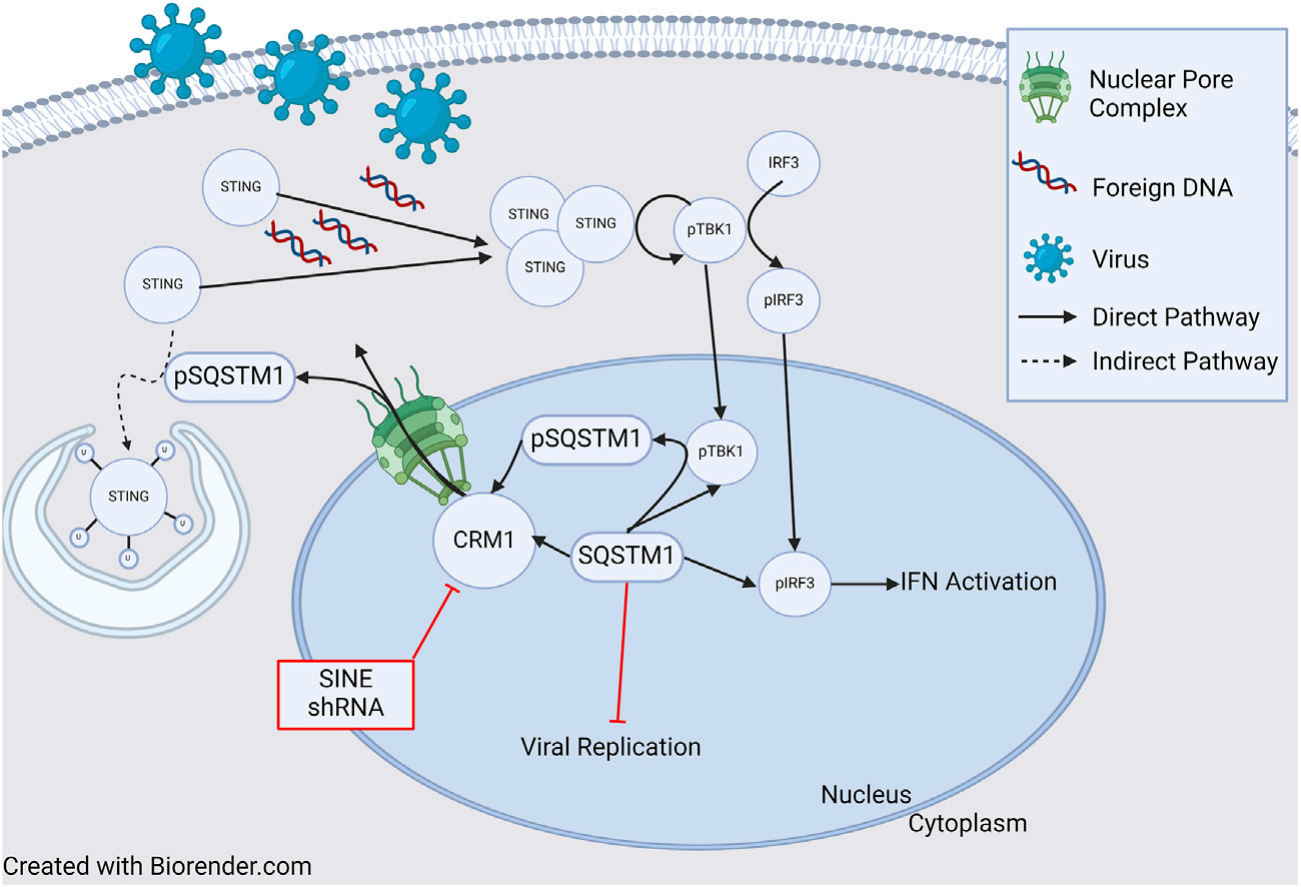
Role of CRM1 and SQSTM1 in Viral Replication and Inflammation. Viral entry into a cell releases foreign DNA which polymerizes STING proteins. The polymerized STINGs induce autophosphorylation of TBK1 which then phosphorylates IRF3. Activated TBK1 and IRF3 (pTBK and pIRF3) then enter the nucleus and induce IFN activation signaling inflammation. In a feedback loop, TBK1 also phosphorylates SQSTM1, which is then shuttled to the cytoplasm in a CRM1 dependent manner, and ultimately (indirectly) ubiquitinates STING, leading to cell STING degradation through the autophagosome. Blockade of SQSTM1 cell exit using either pharmacologic (SINE, or selective inhibitors of nuclear export) or genetic (shRNA) approaches results in increased SQSTM1. Increased SQSTM1 leads to blockade of viral replication, but also activation of IFN-genes through pIRF3, leading to increased inflammation. Figure based on [119–122] and created with BioRender.com.

In addition to its role in HIV, it has been shown that CRM1 is critical for regulation of Kaposi Sarcoma Associated Herpesvirus (KSHV) lytic regulation, and CRM1 inhibition leads to decreased KHSV viral titer. While pharmacologic and genetic (shRNA) inhibition of CRM1 decreased lytic replication, neither had an effect on viral entry and trafficking indicating the important role of CRM1 in viral replication [119]. One potential mechanism of viral replication blockade is through nuclear retention of p62 (SQSTM1) when CRM1 is inactivated. Nuclear retention of SQSTM1 induces activation of TBK1 and IRF3 ultimately activating genes involved in innate immunity through the cGAS/STING pathway. (Figure 3, and further discussion below). Finally, it has been shown that CRM1 is involved in viral life cycle of the parvovirus minute virus of mice, with the virus hijacking host cell machinery and using CRM1 to export critical viral proteins [120].

In summary, many viral replication processes rely on CRM1 and its ability to shuttle RNA or proteins from the nucleus to the cytoplasm. Viruses have been able to take advantage of the CRM1 nuclear export model in simple ways such as directly shuttling proteins out of the nucleus, to more complicated complexes involving dimerization of CRM1 and lncRNAs. Further understanding of viral replication processes may lead to the development of novel therapeutics that may be able to be used in both virology and oncology.

## CRM1 and Inflammation

As previously discussed, there appears to be an influence of CRM1 on the innate immune response and p62 (SQSTM1) through the cGAS/STING pathway. Classically, the cGAS/STING pathway involves the cellular response to foreign DNA. Foreign DNA induces oligomerization of STING (STimulator of INterferon Genes), which activates NF-κB and induces autophosphorylation of TBK1 (TANK Binding Kinase 1), which in turn phosphorylates IRF3. Activated IRF3 (pIRF3) translocates to the nucleus and induces expression of interferons (IFNs), interferon-stimulated genes (ISGs), and other proinflammatory genes (reviewed in [122]). TBK1 also phosphorylates p62/SQSTM1 which is exported from the nucleus and ubiquinates STING leading to its degradation in autophagosomes. Cells deficient in p62/SQSTM1 showed impaired autophagosomal degradation of STING, and in the presence of foreign DNA, this led to an increased amount IFNs [119]. Unsurprisingly, shRNA knockdown of CRM1 and the use of SINEs increased nuclear sequestration of p62/SQSTM1, resulting in increased activity of pIRF3 and pTBK1 and thus, IFNs (Figure 3) [119, 121]. Together, these studies show that through regulation of p62/SQSTM localization, CRM1 has an impact regulating the production of IFNs leading to inflammation.

As discussed above, the CRM1 inhibitor LFS-829 leads to retention of IκB_α_, which inhibits NF-κB transcriptional activation. Other studies using SINEs have also shown decreased expression of NF-κB, which is indicative of the role of CRM1 in regulation of NF-κB expression and ultimately its downstream effectors in the inflammatory system [42]. Additionally, as discussed above, the CRM1 inhibitors SP100030 and SPC-839, also affected the amount of NFAT and subsequently decreased T-cell activation, again indicating the impact of CRM1 on the regulation of inflammation [78].

Much of the work on CRM1’s impact on inflammation has come from the use of CRM1 inhibitors. Thus, this is an area of increased need for understanding of how CRM1 fully impacts inflammation, and ultimately the role of CRM1 and inflammation in other disease process, including oncologic processes.

## Regulation of CRM1 Expression

**FIGURE 4:**
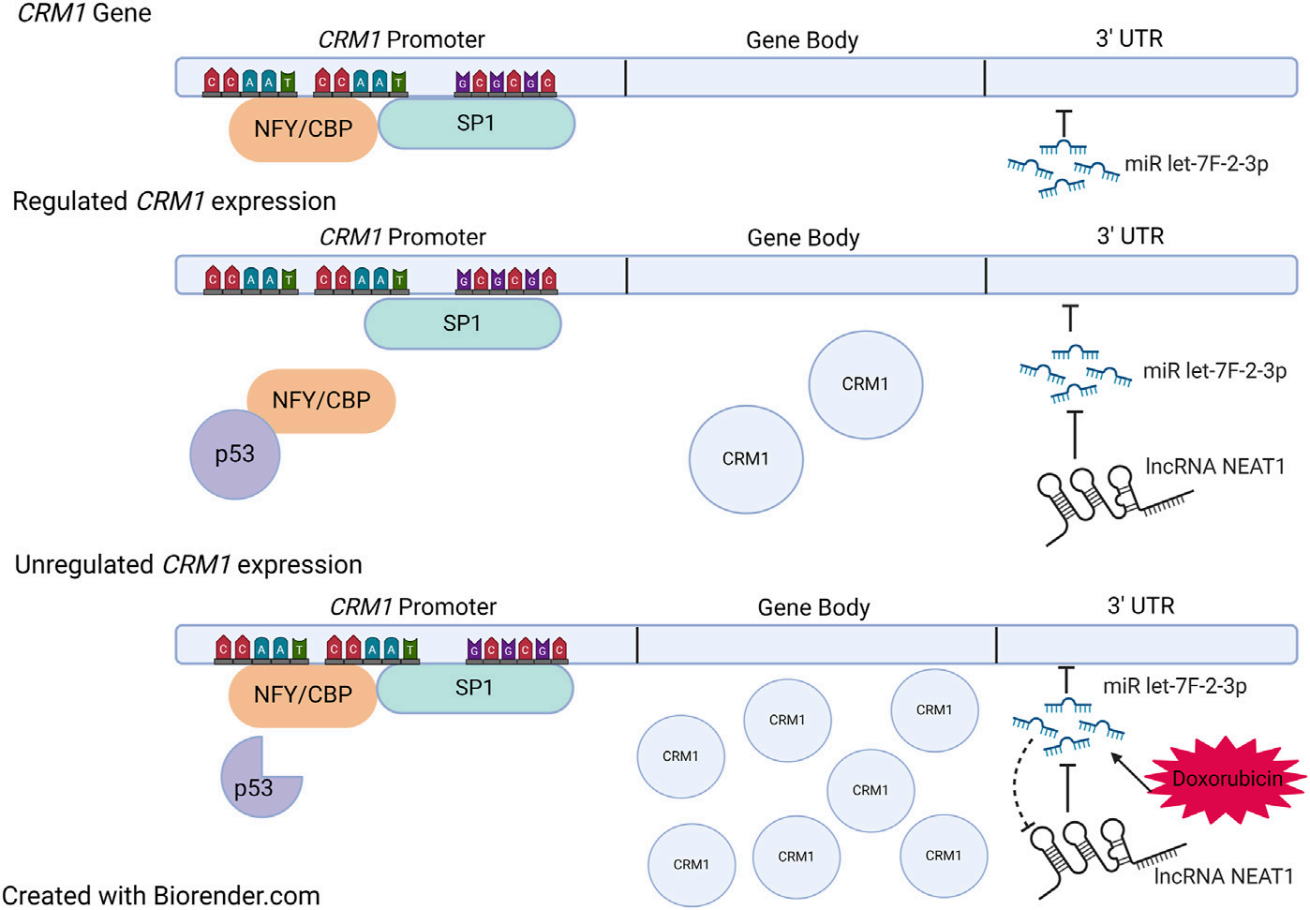
Regulation of *CRM1.* Top: Sites of NFY/CBP, SP1, and miR let-7f-2-3p binding within CRM1. The gene body is under tight regulation from both the promoter region and the 3′ UTR. When NFY/CBP bind the promoter, there is increased expression of CRM1. The binding of NFY/CBP is modulated by p53 in its tumor suppressor role. When p53 is mutated, it no longer binds NFY/CBP, which then allows for increased expression of CRM1. miR let-7f-2-3p downregulates CRM1 expression when bound to the 3′ UTR region, a process which is regulated by the lncRNA NEAT1. Doxorubicin causes increases the expression of miR let-7f-2-3p which then saturates available NEAT1. Ultimately, this leads to decreased presence of the negative regulator of miR let-7f-2-3p, allowing for increased expression of CRM1. Figure based on [123, 124] and created with BioRender.com.

There is evidence that modification of the untranslated regions (UTR) of the CRM1 gene can impact CRM1 protein expression, and more importantly CRM1 protein activity. Specifically, microRNA (miR) let-7f-2-3p has been found to bind the 3′-UTR region of the *CRM1* gene. miR let-7f-2-3p decreases *CRM1* transcription upon binding, a process tightly regulated by the lncRNA NEAT1, discussed above. In general, lncRNAs act as sponges for miRs, and NEAT1, containing miR let-7f-2-3p binding regions, acts by decreasing the available pool of miR let-7f-2-3-p, which ultimately allows for increased CRM1 expression [123] (Figure 4).

Intriguingly, expression of miR let-7f-2-3p is increased by the chemotherapy agent doxorubicin, which is thought to play a role in doxorubicin-induced cardiac toxicity. The increased expression of miR let-7f-2-3p by doxorubicin leads to decreased expression of NEAT1 and thus reduced transcription of *CRM1* mRNA (Figure 4). This leads to nuclear localization of the protein HAX1, a protein that is cardioprotective when cytosolic, yet cardiotoxic when nuclear, evidenced by increased expression myocardial enzyme leakage and activation of cleaved Caspase 3/9 and TUNEL positivity of doxorubicin treated cells. Indeed, introduction of mutations in the miR binding region with the CRM1 3′ UTR region abrogated miR let-7f-2-3p binding, resulting in the absence of HAX1 nuclear localization and myocardial enzyme leak. This mechanism potentially suggests that SINEs could potentially worsen cardiac damage by doxorubicin as a result of increased CRM1 in the nucleus. Despite evidence of synergy between doxorubicin and SINEs, caution should be taken in using these agents together clinically, especially since a Phase Ib trial that combined KPT-330 and doxorubicin in soft tissue sarcomas showed a 20% cardiac adverse event rate (including one grade five event) [125].

The *CRM1* promoter contains two CCAAT boxes and a GC box that are binding sites for the transcription factors NFY/CBP and SP1, which likely play a role in p53-dependent regulation of CRM1 (Figure 4). p53 *represses* CRM1 synthesis, though indirectly. p53 does not directly bind CRM1, instead binding NFY, which effectively removes the activation of CRM1 gene transcription [124]. Thus, it is hypothesized that mutations within p53 may no longer allow NFY-p53 interactions, which would then increase CRM1 expression, potentiating tumorigenesis (Figure 4).

## CRM1 and Cancer

As has been discussed throughout this review, CRM1 is implicated in a number of different physiologic and pathologic processes, including cancer. CRM1’s role in the Hallmarks of Cancer [126] is intimately entrenched in its function as a nuclear exporter, in its role in chromosomal stability, and its potential role in activation of inflammation. Overexpression of CRM1 has been found in a range of cancers from cancers of epithelial/mesenchymal origin such as ovarian, breast, esophageal, prostate, neuroblastoma, lung, gastric, colorectal, melanoma and thyroid to hematologic malignancies such as T-cell lymphoma, chronic lymphocytic leukemia, multiple myeloma, diffuse large B-cell lymphoma, or AML, and increased CRM1 expression leads to a worse prognosis in a number of cancers [1–3, 7, 21, 23, 24, 26–28, 59, 127–131]. Overexpression of CRM1 leads to increased nuclear export; as such, with increased activity of shuttling proteins out of the nucleus, CRM1 is able to *evade growth suppressors* and *sustain proliferative signaling*, two of the Hallmarks of Cancer, by shuttling tumor suppressor genes such as p21, p53, and RB, out of the nucleus, allowing for unchecked growth of the cancer cell and *resistance to cell death,* another Hallmark of Cancer. Thus, the first approach to impacting CRM1-dependent cancers was full inhibition of the protein with the use of the irreversible CRM1 inhibitor Leptomycin B; however, it was quickly determined that too much inhibition leads to death of non-malignant cells due to mislocalization of critical cellular proteins and increased nuclear concentrations of pro-apoptotic proteins. This likely contributed to the failure of Leptomycin B clinically [43]. It is apparent that modifications of the relative amount of functional CRM1 protein, using other drugs such as Selinexor which is partially reversible, can lead to altered cell physiology, allowing for either cell proliferation or cell death.

In addition to overexpression of CRM1, CRM1 has been found to be directly fused to TNRC18 [6] or AF10/MLLT10 [4–6] in AML and T-ALL, respectively. The fusion of CRM1-AF10 has been noted to activate the *HOXA* gene cluster in T-ALL, in a similar manner to CALM-AF10, which requires CRM1 for *HOXA* gene activation [79]. Intriguingly, in this study and others, CRM1 was found to localize to the *HOXA* gene cluster in *CALM-AF10* and *NUP98-HOXA9* fusion leukemias, highlighting its role outside of nuclear export and involvement with DNA and chromatin [80]. The ability of CRM1 to localize directly to DNA may potentially be related to its involvement in chromosomal segregation and organization as discussed above. In addition, overexpression of CRM1 can lead to chromosomal missegregation, and while this typically may lead to apoptosis, it is possible that effects of the abnormal chromosomal separation may lead to *genome instability and mutation*, transforming a normal cell into malignant one, another Hallmark of Cancer.

In addition to overexpression of CRM1 and CRM1 fusing with other proteins found in leukemias, a mutation within CRM1, specifically an E571K mutation has been found in a number of cancers including Chronic Lymphocytic Leukemia, Hodgkins Lymphoma, Primary Mediastinal B-Cell lymphoma, Extra-nodal Natural Killer/T-Cell Lymphoma, and Diffuse Large B-Cell lymphoma [7–10]. In addition to the E571K, 2 other mutations were found in a screen of over 42,000 patients by Taylor, et al [132]. This work showed that mutations of CRM1 induced oncogenesis by altering the nuclear export abilities of CRM1. Interestingly, the mutations within CRM1 did not cause an increase or decrease of nuclear export shuttling *per se*; instead, these mutations altered the ability of proteins to bind to CRM1, which in turn resulted in altered nuclear versus cytoplasmic localization of proteins. For instance, nuclear export of the protein TRAF2 was increased in the cells harboring the E571K mutation of CRM1, whereas there was decreased shuttling of p120 catenin in the same cells. This group showed that cargo that contain a negatively charged C-terminus NES sequence, such as is found in TRAF2, will undergo more extensive nuclear export, whereas the converse is true for cargoes with a positively charged C-terminus.

Given the role of CRM1 in cancer, it is then not surprising that CRM1 inhibitors have been investigated as potential treatment options, both as single agents and in combination with other chemotherapeutic options. Tables 1, 2 review clinical trials that, as of this writing, have used CRM1 inhibitors that are either open (Table 1) or closed (Table 2). With these ongoing clinical trials, it will be important to gain further understanding of the cellular impact of these inhibitors. Studying the nuclear/cytoplasmic localization of proteins following treatment with CRM1 inhibitors, can potentially lead to the identification of activated pathways that may be targeted with small molecule inhibitors, either novel or repurposed such as what happened with Zafirlukast, to proteins involved in these pathways that could be then used in cancer treatments.

## Discussion

While its most widely studied role is in nuclear export, CRM1 is also involved in other cellular processes, including cell replication via centromere and centrosome formation as well as viral replication. Because of its critical cellular functions, its expression is also tightly regulated from a transcriptional standpoint, and also as a result of distinct structural domains and post-transcriptional modification. The importance of CRM1 is also evidenced by viral hijacking–viruses have evolved to use CRM1 to perpetuate their own replication and shuttling of viral RNA from the nucleus to the cytoplasm.

Since protein localization aberrations are often found in malignancies, it is not surprising that CRM1 is either directly or indirectly involved in oncogenesis, and that inhibiting CRM1 may lead to tumor cell death. A deeper understanding of mechanisms of CRM1 activity will allow for the discovery of novel inhibitors, with the goal of fewer side effects. For example, finding a small molecule inhibitor of the NUP/CRM1 interaction could prevent binding of CRM1 to NUP containing oncoproteins which might allow for restoration of its nuclear export activity. Further understanding the mechanisms of CRM1 interaction with RBM14, SUN1/SUN2, p54nrb, NPM1, HOPS, γ-tubulin or eEF-1A will allow for the development of other targeted therapies for these interactions. Finding inhibitors that bind to sites within the UTR regions of the *CRM1* gene will have the benefit of minimizing off-target effects.

While the majority of work describing CRM1 relates to its role as a nuclear export protein, it is clear that CRM1 is involved in other critical cellular processes including chromosomal organization, inflammation, and its mechanisms are hijacked by both viruses and cancer cells (Table 4). A better understanding of the CRM1 protein, its mechanism of action, and regulation of its expression should lead to the ability to develop new therapeutics allowing for precise modifications CRM1 and its activity.

**TABLE 4:** Summary of CRM1 functions and effect of increased CRM1 expression or inhibition.

	Normal function	Increased CRM1 expression	Inhibited CRM1
Nuclear Export	Export proteins from nucleus to cytoplasm	Abnormal shuttling of proteins to cytoplasm	Abnormal localization of proteins to nucleus
Inflammation	Regulation of IFN production through p62 (SQSTM1) localization regulation	Future area of research	Nuclear retention of p62 (SQSTM1) leads to impaired STING autophagosomal STING degradation and increased IFNs Nuclear retention of IκB_α_, which inhibits NF-κB activation Decreased T-Cell activation
Viral Pathogenesis	Export of viral RNA	Increased viral replication	Decreased viral replication through nuclear retention of p62 (SQSTM1)
Chromosomal Organization	Development of single centromere and centrosome duplication	Premature microtubule attachment to spindle leading to abnormal chromosomal separation during mitosis	Abnormal duplication of centrosomes leading to disordered chromosomal segregation

## Abbreviations

AML, Acute Myeloid Leukemia; CRM1, Chromosome Region Maintenance 1; FG, Phenylalanine/Glycine; FG-NUPS, Nucleoporins with phenylalanine/glycine repeats; HEAT, Huntingtin, Elongation factor 3, Alpha subunit of protein phosphatase 2A, and the TOR1 PI3 kinase; IFN, Interferon; ISG, Interferon-Stimulated Genes; LFS, *Lai Fu Zi* (*Raphanus sativus*); LINC, Linker of Nucleoskeleton and Cytoskeleton; LMB, Leptomycin B; NCI-1, Non-covalent CRM1 Inhibitor -1; NES, Nuclear Export Sequence; NPC, Nuclear Pore Complex; NUP, Nucleoporin; RRE, Rev Response Element; STING, STimulator of INterferon Genes; XPO1, Exportin 1.
